# Rapid Quantification of SARS-Cov-2 Spike Protein Enhanced with a Machine Learning Technique Integrated in a Smart and Portable Immunosensor

**DOI:** 10.3390/bios12060426

**Published:** 2022-06-17

**Authors:** Simone Fortunati, Chiara Giliberti, Marco Giannetto, Angelo Bolchi, Davide Ferrari, Gaetano Donofrio, Valentina Bianchi, Andrea Boni, Ilaria De Munari, Maria Careri

**Affiliations:** 1 Dipartimento di Scienze Chimiche, della Vita e della Sostenibilità Ambientale, Università di Parma, Parco Area delle Scienze 17/A, 43124 Parma, Italy; simone.fortunati@unipr.it (S.F.); chiara.giliberti@unipr.it (C.G.); angelo.bolchi@unipr.it (A.B.); davide.ferrari@unipr.it (D.F.); 2 Dipartimento di Scienze Medico-Veterinarie, Università di Parma, Strada del Taglio 10, 43126 Parma, Italy; gaetano.donofrio@unipr.it; 3 Dipartimento di Ingegneria e Architettura, Università di Parma, Parco Area delle Scienze 181/A, 43124 Parma, Italy; valentina.bianchi@unipr.it (V.B.); andrea.boni@unipr.it (A.B.); ilaria.demunari@unipr.it (I.D.M.)

**Keywords:** machine learning, SARS-CoV-2, COVID-19, electrochemical immunosensor, IoT-WiFi, point of care testing, gold nanoparticles

## Abstract

An IoT-WiFi smart and portable electrochemical immunosensor for the quantification of SARS-CoV-2 spike protein was developed with integrated machine learning features. The immunoenzymatic sensor is based on the immobilization of monoclonal antibodies directed at the SARS-CoV-2 S1 subunit on Screen-Printed Electrodes functionalized with gold nanoparticles. The analytical protocol involves a single-step sample incubation. Immunosensor performance was validated in a viral transfer medium which is commonly used for the desorption of nasopharyngeal swabs. Remarkable specificity of the response was demonstrated by testing H1N1 Hemagglutinin from swine-origin influenza A virus and Spike Protein S1 from Middle East respiratory syndrome coronavirus. Machine learning was successfully used for data processing and analysis. Different support vector machine classifiers were evaluated, proving that algorithms affect the classifier accuracy. The test accuracy of the best classification model in terms of true positive/true negative sample classification was 97.3%. In addition, the ML algorithm can be easily integrated into cloud-based portable Wi-Fi devices. Finally, the immunosensor was successfully tested using a third generation replicating incompetent lentiviral vector pseudotyped with SARS-CoV-2 spike glycoprotein, thus proving the applicability of the immunosensor to whole virus detection.

## 1. Introduction

In recent years, there has been a marked increase in interest toward the so-called Internet of Things (IoT) paradigm in both the academic [1] and industrial [2] contexts. IoT-compliant sensors and systems are spreading in several fields, facilitating data collection and sharing, ensuring ubiquitous applications and overcoming the use of specialized environments (e.g., laboratories, industries, etc.). The opportunities offered by IoT devices are being further expanded by the ability to combine IoT with Machine Learning (ML) algorithms [3]. Through ML, a system model can be automatically created from a known labeled data set and can be used to predict the output of the system when new input sets associated with unknown outputs are provided. The features offered by ML represent a great advantage in several fields, especially in relation to the management of infectious diseases [4,5,6,7]. A clear example can be found in the pandemic caused by the SARS-CoV-2 virus [8,9,10,11]. The rapid spread of the virus and the lack of effective treatments for the disease have led to millions of deaths worldwide, as well as severe socioeconomic consequences. In this currently ongoing scenario, a pivotal role is played by diagnostic methods for the efficient tracking of the disease and swift response after the identification of clusters of infections in specific areas. To this end, the availability of diagnostic methods, which offer a fast and reliable detection of the virus in clinical samples, plays a crucial role. Furthermore, point-of-care testing (PoCT) features that allow decentralized diagnostic analyses are required to reach remote areas.

Regarding the diagnosis of SARS-CoV-2 infection in humans, the gold standard at present is the detection of SARS-CoV-2 viral RNA in nasopharyngeal swabs using polymerase chain reaction (PCR)-based techniques. Notwithstanding the accuracy in virus detection, these molecular assays suffer from some drawbacks, including long time to results, the risk of low specificity through cross-contamination, the need for highly skilled personnel and the need to perform analyses in centralized laboratories [12,13]. Alongside PCR techniques, lateral-flow immunoassays (LFIA) offer a rapid, portable, low-cost and easy-to-use alternative for the detection of SARS-CoV-2-related antigens, mainly nucleocapsid protein and spike (S) protein. On the other hand, the accuracy of LFIA tests in terms of sensitivity and specificity is generally low. Additionally, they do not offer quantitative data and the interpretation of the results can be ambiguous for antigen concentrations approaching the limit of detection (LOD) [14,15,16].

To overcome these limitations, the scientific community has focused on the engineering and development of innovative biosensors, which offer remarkable versatility in terms of target species that can be quantified. In fact, by applying a suitable biomolecule to be used as a receptor, it is possible to target mainly antibodies [17,18,19,20], antigens [21,22,23,24] or oligonucleotides [25,26,27,28]. Furthermore, biosensing methods are usually rapid, implemented in portable and cost-effective analytical devices as well as in smart PoCT devices for analytical signal acquisition. In the case of the detection of SARS-CoV-2 virus in patient samples, several biosensing techniques have been proposed based on different signal transduction mechanisms, including optical [29,30], electrical [31,32,33] and electrochemical ones [34,35,36,37,38]. Among these, Fabiani et al. reported the development of an immunoassay based on the use of magnetic beads and carbon black-modified screen-printed electrodes for the sensitive detection of both the nucleocapsid and spike proteins of the SARS-CoV-2 virus in saliva samples using a commercial, portable potentiostat with Bluetooth connectivity [39]. The authors demonstrated the ability to detect the spike protein in untreated saliva with a detection limit of 19 ng/mL and to correctly classify 22/24 clinical samples. However, it is worth pointing out that the use of magnetic beads for the execution of the assay requires dedicated equipment for their isolation and washing, which could limit the applicability of the method in decentralized settings. Recently, Beduk et al. [40] reported a label-free electrochemical immunoassay based on laser-scribed graphene electrodes for the detection of S1 subunit of SARS-CoV-2 spike protein using a custom-made PoCT device with Bluetooth connectivity. The platform was applied to 23 blood serum samples of COVID-19 patients for clinical validation, with a LOD of 2.9 ng/mL in buffer solution; however, even though possible interference from immunoglobulins was assessed, the responses generated by antigen proteins from other frequently occurring viruses were not evaluated. More recently, the same authors improved the performance of the developed device by including machine learning (ML) features and by replacing the antibody receptor with a human ACE2 enzyme [8], enabling the evaluation of the response induced by different variants of SARS-CoV-2 in nasopharyngeal swab samples. In this, case a LOD of 5.14 ng/mL was obtained for SARS-CoV-2 S1 with a sample incubation time of one hour; however, the developed biosensor showed moderate selectivity towards SARS-CoV-2 among other common respiratory viruses, such as influenza A H1N1.

**Figure 1:**
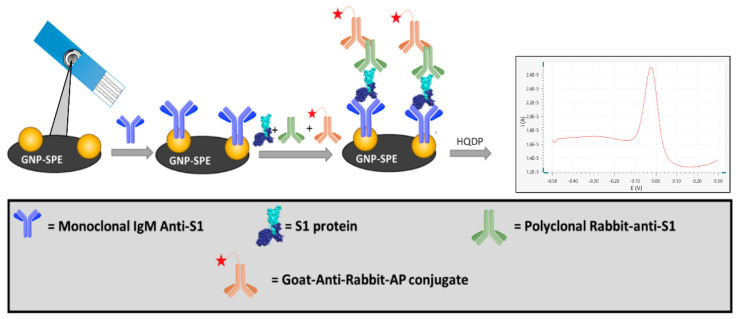
Schematic illustration of the protocol used for the development of the electrochemical immunosensor for the quantification of S1 subunit of SARS-CoV-2 spike protein.

In the present study, we developed an innovative electrochemical immunosensor based on screen-printed electrodes functionalized with gold nanoparticles (GNP-SPEs) for the quantification of the S1 subunit of the SARS-CoV-2 spike protein. The biosensor format using an immunoenzymatic approach is based on a sandwich protocol involving a monoclonal antibody as a receptor (capture antibody), an anti-S1 polyclonal antibody, and an alkaline phosphatase-conjugated secondary antibody as reading antibodies (Figure 1).

Analytical validation was performed in a Viral Transfer Medium (VTM) matrix used for the desorption of nasopharyngeal swabs, showing high performance in terms of specificity with respect to other viral proteins of pathogens, i.e., MERS-CoV and H1N1, which are potentially present in clinical samples. Furthermore, the new immunosensor was tested on lentiviruses that express the SARS-CoV-2 spike protein on their surface, thus demonstrating its ability to detect the whole virus in nasopharyngeal swab tests.

The immunosensor was interfaced with a WiFi-based portable device for signal readout and on-cloud data processing which enables ML features [41]. This IoT portable potentiostat was connected through a Wi-Fi protocol with the Thingspeak cloud service, which consists of an IoT data analytics platform with the ability to use MATLAB for data analyses [42]. The choice of this platform was therefore aimed at integrating ML techniques into a single solution that does not require additional external platforms or devices.

We demonstrated that ML techniques are suitable for classifying true positive and true negative samples with high accuracy (>90%), without the need for any pre-processing of the data acquired by the immunosensor. Moreover, the ML techniques are capable of processing the voltammograms acquired through Differential Pulse Voltammetry (DPV) over a wider potential range than the traditional calculation of the current peak. This results in a considerable simplification of both the acquisition set-up of and the on-board processing. In fact, the training phase, which has to be carried out only once, can be performed on the cloud platform, taking advantage of a higher computation power than that available on a microcontroller unit (MCU) platform. The obtained training model can be automatically coded in the C language and implemented on the MCU platform. Therefore, the processing of the data acquired during normal operation (test phase) is carried out on board, thus simplifying the test process even for users without particular technical skills. By limiting the computational power and the time required during the test phase, we were able to implement the whole acquisition and processing actions on a low-cost, low power MCU platform with benefits in terms of flexibility (no need to select a particular platform) and a higher usability (longer battery life, ease of use). Even though a similar approach was applied recently by Beduk et al. [8], that solution requires external devices for the training phase and a connection via a wired serial port, limiting portability. Moreover, that device is powered via a smartphone USB connection. The smartphone is also needed as a gateway to the internet or for local data visualization, since the wireless connection relies on the Bluetooth protocol. Additionally, traditional preprocessing is performed to evaluate the DPV current peak, with a baseline computation from the patient sample.

We used a Support Vector Machine (SVM)-based classifier to automatically label positive and negative samples. This method may achieve higher accuracy than traditional semi-quantitative analysis based on the LOD threshold [19]. SVM belongs to a class of supervised algorithms [43] which are capable of recognizing a specific pattern once trained using a set of labeled data to optimize their separation and assign them to the correct class by means of suitable kernel function. To this end, different kernel methods can be selected according to the mathematical function used in the data separation (e.g., linear, quadratic, cubic, gaussian etc.). The parameters of the trained model can be optimized to achieve even higher accuracies.

In this context, our PoCT sensing device, implemented on a portable, wireless and standalone instrumentation, has proved to be suitable for the integration of ML algorithms to improve the accuracy in the classification of responses for the detection of the SARS-CoV-2 spike protein.

## 2. Materials and Methods

### 2.1. Materials and Chemicals 

Sodium chloride (NaCl), potassium chloride (KCl), potassium dihydrogen phosphate (KH_2_PO_4_), di-sodium hydrogen phosphate (Na_2_HPO_4_), Trizma^®^ Base, magnesium chloride (MgCl_2_), *N*-(3-dimethyl)-*N*’-ethylcarbodiimide hydrochloride (EDC), *N*-Hydroxysuccinimide (NHS), 4-morpholineethanesulfonic acid monohydrate (MES), Tween^®^ 20, bovine serum albumin (BSA) were purchased from Merck (Milan, Italy). SARS-CoV-2 S1 subunit, SARS-CoV-2 Spike S1 Antibody (hIgM2001; mA-S1), Human Chimeric and H1N1 (A/California/04/2009) Hemagglutinin (HA) were purchased from GenScript (Piscataway, NJ, USA).

SARS-CoV-2 Spike Protein S1 Polyclonal antibody (pA-S1) and Goat anti-Rabbit IgG secondary antibody conjugated with alkaline phosphatase (GAR-AP) were purchased from Invitrogen, ThermoFisher Scientific (Waltham, MA, USA). Viral Transport Medium (VTM) with CDC formula was purchased from Capricorn Scientific (Ebsdorfergrund, Germany).

Recombinant Coronavirus Spike Protein MERS-CoV-S1 was purchased from Biovision (Milpitas, CA, USA).

Hydroquinone diphosphate (DRP HQDP) was purchased from Metrohm Italiana s.r.l. (Origgio, Italy).

p8.74 packaging and pREV pseudotyping vectors were obtained from Addgene (Teddington, UK). DMEM was purchased from Euroclone s.p.a. (Milan, Italy). Eagle’s Minimal Essential Medium (EMEM) and fetal bovine serum were purchased from Gibco, Thermo Fisher Scientific (Carlsbad, CA, USA). Polyethylenimine (PEI) transfection reagent was from Polysciences, Inc. (Warrington, PA, USA). 0.45 μm filters were obtained from Millipore, Merk (Darmstadt, Germany).

HEK 293T cells (CRL-1573) were from American Type Culture Collection (ATCC, Manassas, VA, USA).

### 2.2. Buffers Composition 

Phosphate Buffer Saline (PBS) at pH = 7.4 was prepared in distilled water with the following composition: 137 mM NaCl, 2.7 mM KCl, 1.2 mM KH_2_PO_4_, 8 mM Na_2_HPO_4_. PBS-t was prepared by addition of the surfactant Tween^®^ 20 to PBS to reach a final concentration of 0.05% (*w*/*v*).

TRIS buffer at pH = 7.4 and reading buffer at pH = 9.8 were prepared in distilled water with the following composition: 0.1 M Trizma Base, 0.02 M MgCl_2_. TRIS-t was prepared by addition of the surfactant Tween^®^ 20 to TRIS buffer to reach a final concentration of 0.05% (*w*/*v*).

MES buffer at pH = 5.0 was prepared in distilled water according to the following composition: 1.06 g of MES.

### 2.3. Equipment

Immunosensors were assembled on single-walled carbon nanotubes-modified screen-printed carbon electrodes (SWCNT-SPEs; DropSens DRP-110SWCNT) and gold nanoparticles-modified screen-printed carbon electrodes (GNP-SPEs; DropSens DRP-110GNP), purchased from Metrohm Italiana Srl (Origgio, Varese, Italy). The size of the SPEs was 3.4 × 1.0 × 0.05 cm, with a working electrode diameter of 4 mm. Reference electrode and electric contacts were made of silver, whereas the counter electrode was made of carbon. All electrochemical measurements were performed using the smart potentiostat developed in this work. 

The smart potentiostat described in [41] has proven to be suitable for data acquisition. It is a portable wireless device that uses the Wi-Fi protocol to connect to a cloud environment for data processing and visualization. The main feature of this device is that, unlike the solutions presented in the literature [8], it does not need to rely on external devices (e.g., PC, tablets or smartphones) for operation. In addition to greatly simplifying the system architecture, this feature makes the device particularly suitable for PoCT applications.

**Figure 2:**
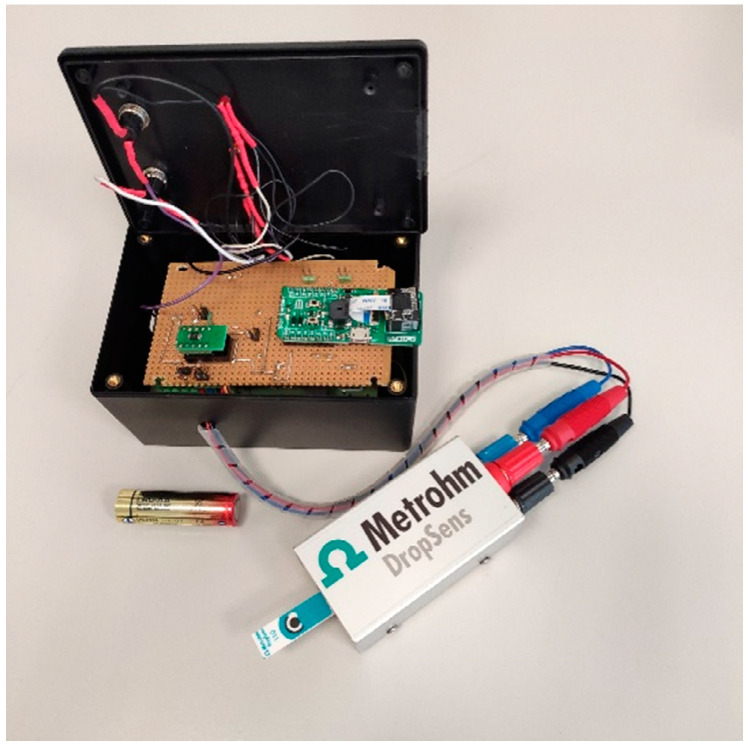
The smart portable wireless potentiostat.

The device, which is shown in Figure 2, consists of a custom Analog Front End (AFE) interfaced with a microcontroller that embeds a Wi-Fi network processor (i.e., CC3200 by Texas Instruments) [41]. Its dimensions are 12 × 8 × 6 cm (LxWxH) for a weight of 200 g. The device can be powered by two 1.5V AA batteries.

The AFE is designed with discrete components and includes a TransImpedance Amplifier (TIA) with a programmable gain to sense the cell current, a 16-bit Digital to Analog Converter (DAC) to generate the conditioning potentials and a 14-bit Analog to Digital Converter (ADC) to acquire data and send them to the microcontroller. The implementation is intended to minimize the number of required components and the power consumption. Considering the case of five acquisitions per day and two 1.5 V, 2700 mAh AA batteries, an autonomy of about 3.8 years can be achieved. A barcode reader is interfaced with the serial port of the microcontroller to match the personal ID with the acquired data. Samples can be processed on the edge in order to reduce energy consumption, or sent to a cloud environment where they can be processed to take advantage of higher computing power.

Hybrid solutions can also be implemented with onboard pre-processing. The cloud service selected is ThingSpeak, an IoT analytics platform based on a MATLAB engine, although connection to other services can be implemented. Thingspeak is also used for the configuration, storage and visualization of data thanks to an integrated custom web application. Thanks to the Wi-Fi connection, no Internet gateway or dedicated network infrastructure is required. It is worth noting that thanks to the possibility of performing data processing either on the local microcontroller or the remote cloud service, it is possible to choose the best compromise between higher computing power or lower energy consumption, depending on the considered application. For instance, it is possible to perform complex and demanding computations such as training a ML algorithm completely autonomously from third-party devices or software, obtaining a compact, integrated and flexible approach which is particularly suitable for a PoCT context.

### 2.4. Immunoassay Setup

SARS-CoV-2 Spike S1 Antibodies were diluted in PBS buffer to a final concentration of 15 µg/mL and 25 µL of solution were drop casted on the GNP-SPE overnight at +4 °C. After chemisorption of the receptor on GNP substrate, electrodes were rinsed with PBS-t and PBS buffer.

To prevent nonspecific binding, a blocking treatment was carried out by drop casting 50 µL of a 20 mg/mL solution of BSA dissolved in PBS. After 30 min of incubation, the electrodes were rinsed with TRIS-t and TRIS buffer.

The SARS-CoV-2 S1 protein was diluted by a selected factor (see Section 3.2.) in a solution containing TRIS and Viral Transport Medium in a 1:1 ratio; then, this solution was mixed with a previously prepared solution containing pA-S1 and GAR-AP to reach a final concentration of 1 µg/mL and 2 µg/mL respectively. Twenty-five microliters of the resulting solution were drop casted on each electrode and incubated for 1h, after which a washing step with TRIS-t and TRIS was performed.

The readout step was carried out by drop-casting 50 µL of a 1 mg/mL solution of HQDP dissolved in reading buffer and, after 150 s of incubation of the enzymatic substrate, a DPV measurement was performed between −0.5 V and +0.1 V (step potential = + 0.00495 V, modulation amplitude = + 0.04995 V, modulation time = 0.102 s, interval time = 0.4 s).

### 2.5. Experimental Design

A Full Factorial Design (FFD) with two factors and three levels (3^2^) was carried out [19]. The response variable was *P*/*N* (*P* = positive sample; *N* = negative sample) to obtain the best concentration of SARS-CoV-2 Spike S1 IgM Antibody (mA-S1) and SARS-CoV-2 Spike Protein S1 Polyclonal antibody (pA-S1).

ANOVA tests were carried out to evaluate the significance of two factors. Each of the experiments was replicated three times and acquired under randomized sequence. All statistical calculations were performed using the Statistics for Data Analysis V.28 software package (SPS Srl, Bologna, Italy).

### 2.6. Analytical Validation

Validation of the developed immunosensor was performed according to Eurachem Guide [44]. Measurements were carried out on solutions containing 1:1 diluted VTM spiked with SARS-CoV-2 S1 subunit at different concentrations. To achieve this, LOD, limit of quantification (LOQ), linearity and precision in terms of repeatability were calculated performing three replicated measurements for each level explored. As for LOD and LOQ, 10 replicate measurements of blank samples, i.e., matrices containing no detectable analyte, were carried out. LOD was calculated as 3·$s_{0}$/$\sqrt{n}$ and LOQ as 10·$s_{0}$/$\sqrt{n}$, where $s_{0}$ is the blank standard deviation and *n* is the number of replicate measurements. Concerning the evaluation of linearity, regression residuals were calculated, the mean of which over the linearity range was not significantly different from zero (*p* > 0.05). Precision was measured on two levels, i.e., lower and upper levels of the calibration curve and expressed as Relative Standard Deviation (RSD).

### 2.7. SARS-CoV-2 Pseudovirus Generation 

Lentiviral vector-based SARS-CoV-2 spike pseudoviruses were generated as previously described [45] with minor modifications. Briefly, HEK 293T cells were transfected in T175 cm^2^ flasks with:

- 25 μg of pLV-EF1α-(turboGFP-Luc2)-WPRE transfer vector
- 15 μg of p8.74 packaging vector
- 13 μg of pseudotyping vector coding for spike glycoprotein [Wuhan-Hu-1 (B.1 Lineage; China)]
- 5 μg of pREV

The mixture (58 μg of total DNA) was diluted in 3 mL of complete DMEM without serum, and 145 μL of 1 mg/mL PEI in PBS was added (ratio DNA/PEI 1:2.5).

After at least 15 min incubation at room temperature, 4 × volumes of complete DMEM without serum were added, and the transfection solution was transferred to the cell monolayer. After 6 h of incubation at 37 °C and 5% CO_2_ in a humidified incubator, the transfection mixture was replaced with 25 mL of fresh complete EMEM supplemented with 10% FBS and incubated for 48 h at 37 °C and 5% CO_2_. The flask was then frozen–thawed at −80 °C; transfected cell supernatant (TCS) containing spike pseudovirus was clarified via centrifugation at 3500 rpm for 5 min at 4 °C, filtered through a 0.45 μm filter, aliquoted, titered by limited dilution, and stored at −80 °C.

## 3. Results and Discussion

### 3.1. Immunosensor Setup

Taking into account that the present study aims at the development of an immunosensor for the detection of S1 protein, which is a subunit of the Spike protein expressed on the surface of Sars-CoV-2 virus, we devised an electrochemical immunosensor based on the immobilization of mA-S1 used as receptor on the surface of commercially available screen-printed electrodes. As proven in our previous studies [22], different nanostructured materials embedded on the electrode inks of SPEs allow to achieve an increase in the active surface, determining an increase in the performance of the immunosensor. For this purpose, we compared SPEs modified with gold nanoparticles and single-walled carbon nanotubes in terms of receptor immobilization efficiencies on the different nanostructures. After functionalization of SPE with mA-S1, the determination of SARS-CoV-2 S1 subunit was performed using a single-step protocol in which the sample was first mixed with a solution containing both pA-S1 and GAR-AP and subsequently incubated on the electrode surface. This solution also contains Viral Transport Medium, which is commonly used for desorption of samples collected via nasopharyngeal swabs.

As already reported [39], in a sample containing the antigen, pA-S1 antibodies specifically bind several epitopes of the target SARS-CoV-2 S1, while the Fc fraction of pA-S1 is recognized by the GAR-AP secondary antibody through which the electrochemical signal is generated. Concurrently, the mA-S1 antibody will bind the entire immune complex on the SPE via a specific epitope of the viral protein subunit. The use of a polyclonal antibody instead of a conventionally used monoclonal antibody for sandwich immunoassays results in a signal amplification with a consequent sensitivity enhancement.

Alkaline phosphatase was used to detect immuno-absorbed antigens by the addition of the HQDP substrate [19,21,22,23]. The resulting peak intensity acquired by means of DPV was proportional to the amount of GAR-AP and, in turn, of the SARS-CoV-2 S1 subunit bound to the receptor. DPV analysis was performed using the smart portable potentiostat previously developed and described in [41], where a performance equivalent to that of a commercial benchtop potentiostat was proven.

### 3.2. Immunosensor Optimization and Assessment of Sensitivity and Specificity

In order to determine the best conditions for the execution of the electrochemical immunoassay, the electrode substrate and the mA-S1 capture antibody isotype were investigated. Regarding the SPE modification, sensor performance was compared using GNP-SPEs and SWCNT-SPEs. It is worth pointing out that the SPE substrate not only affects the electrochemical properties of the system, but also determines the portion of the bioreceptor involved in the immobilization stage, and therefore, its orientation [22]. In fact, the immobilization on SWCNT is performed through a coupling reaction between the lysine moieties of the capture antibody and the carboxylic functionalities of SWCNT-SPE to generate a covalent amide bond. Conversely, the direct chemisorption of the cysteine residues of the capture antibody on gold is exploited for the functionalization of GNP-SPE with mA-S1 in such a way that, based on the selected electrode substrate, the receptor assumes a specific orientation which will affect its availability for interaction with the target protein.

**Figure 3:**
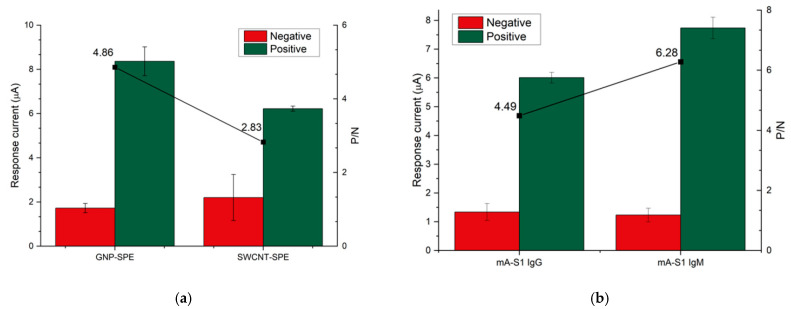
Effect of (**a**) electrode substrate and (**b**) monoclonal capture antibody isotype on the response and *P*/*N* ratio of the immunosensor.

To assess performance using the two aforementioned nanostructured electrode substrates, mA-S1-IgG was immobilized on both SWCNT- and GNP-SPE at a concentration of 10 µg/mL, setting pA-S1 and GAR-AP concentrations at 10 µg/mL and 2 µg/mL, respectively. Positive signals were acquired using SARS-CoV-2 S1 at a concentration of 15 µg/mL, while blank solutions were used as negative samples (Figure 3a). The best ratio between positive and negative signals (*P*/*N*), i.e., 4.86, was obtained using GNP-SPEs, suggesting that cysteine residues of mA-S1, used for immobilization on GNPs, are less involved in the interaction with SARS-CoV-2 S1 subunit.

In order to further improve the sensitivity of the immunosensor, two isotypes of monoclonal receptor antibodies, namely IgG and IgM, were compared (Figure 3b). Both IgG and IgM mA-S1 were used at a concentration of 10 µg/mL, while pA-S1 and GAR-AP concentrations were set at 10 µg/mL and 2 µg/mL, respectively.

The results show that IgM mA-S1 antibodies both improved the sensitivity and achieved better discrimination between positive and negative signals than IgG mA-S1. The better performance of monoclonal IgM compared to IgG can be attributed to various factors: (i) after immobilization on the SPE, IgM may retain more sites for antigen binding as more epitopes, i.e., ten instead of two, are available compared to IgG; (ii) the epitope of the S1 protein within the immune complex recognized by IgM, which is different from that recognized by IgG, may be less masked by the pA-S1 antibodies.

Based on these findings, IgM mA-S1 was selected as the capture antibody and GNP-SPEs were chosen as the platform for performing the electrochemical immunoassay.

In order to assess the effective immobilization of IgM mA-S1, targeted experiments were carried out on GNP-SPEs not functionalized with the monoclonal capture antibody, incubating a 15 µg/mL solution of SARS-CoV-2 S1 also containing pA-S1 and GAR-AP at 10 and 2 µg/mL, respectively. A signal not significantly different (*p* > 0.05) from blank was obtained, thus proving both the effectiveness of the immobilized IgM mA-S1 and the absence of non-specific binding. The results of these experiments are reported in the Supplementary Materials (Figure S1).

After selecting the electrode substrate and receptor isotype, a three-level, two-factor Full Factorial Design involving mA-S1 IgM and pA-S1 concentrations (factor A and B, respectively) was applied to investigate the influence of these experimental factors and their interaction effects on the response, i.e., the ratio between positive and negative samples (*P/N*).

Three different concentrations were explored for each factor, namely 1, 5, 10 µg/mL for pA-S1 and 5, 10, 15 µg/mL for mA-S1 IgM. A two-way ANOVA with Bonferroni post-hoc test was performed to confirm statistical significance for mA-S1 IgM (*p* < 0.01), pA-S1 (*p* < 0.05) effects and their interaction (*p* < 0.01).

**Figure 4:**
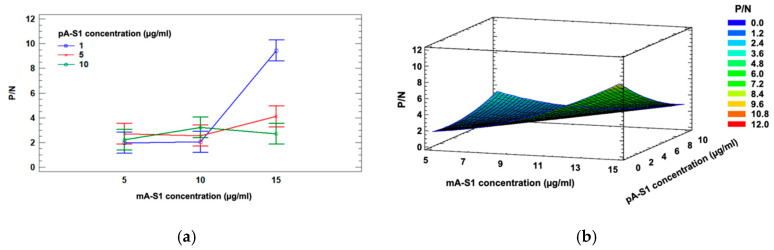
ANOVA interaction plot (**a**) and response surface (**b**) showing the effect of pA-S1 and mA-S1 on the *P*/*N* ratio.

A significant interaction between the factors is also highlighted in the ANOVA interaction plot (Figure 4a).

The mathematical model which best fit to the data was the quadratic model reported in Equation (1); the corresponding response surface is shown in Figure 4b.

*P/N* = 2.0231 − 0.289467·A + 0.235617·B + 0.0501·A^2^ − 0.0751311·A·B+ 0.0289352·B^2^ (1)

Considering the aforementioned experimental data, the optimal response value estimated from Equation (1) was *P*/*N* = 8.09, corresponding to the following optimal parameters: 1 µg/mL for pA-S1 and 15 µg/mL for mA-S1.

The analytical performance of the immunosensor was then evaluated under the optimized experimental conditions by performing measurements in VTM in order to simulate specimens collected from patients, since VTM is commonly used for desorption of nasopharyngeal swabs. Given its complexity, this matrix is known to generate interference in analytical responses [28]. In particular, its composition includes a physiologically balanced isotonic buffered solution at neutral pH, antibacterial and antifungal agents, fetal bovine serum proteins as well as disinfectants. 

**Figure 5:**
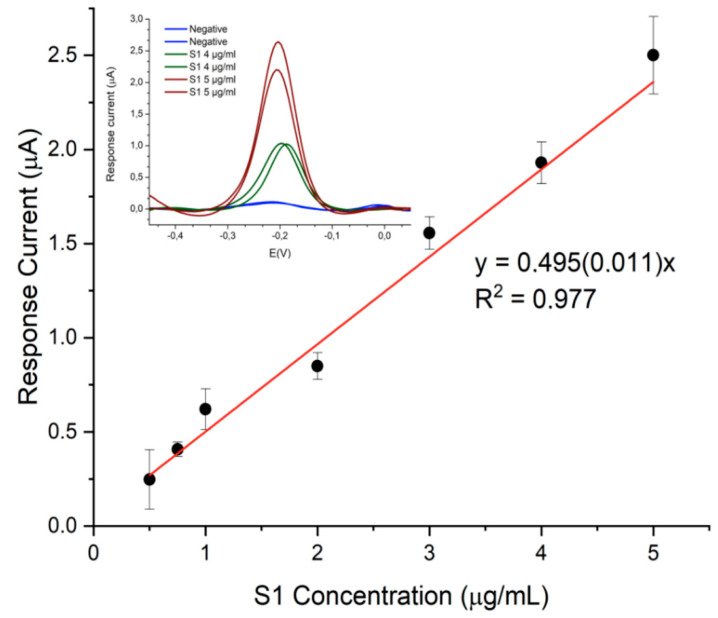
Calibration curve of the immunosensor for SARS-CoV-2 S1 protein. Inset: DPV responses acquired using the portable potentiostat.

The designed immunosensor displayed good analytical performance in terms of sensitivity, precision and specificity. Exploring the range between 0,25 µg/mL and 10 µg/mL of S1 protein, linearity was observed in the 0.5–5 µg/mL range (Figure 5) with detection and quantification limits of 12 ng/mL and 40 ng/mL, respectively.

**Figure 6:**
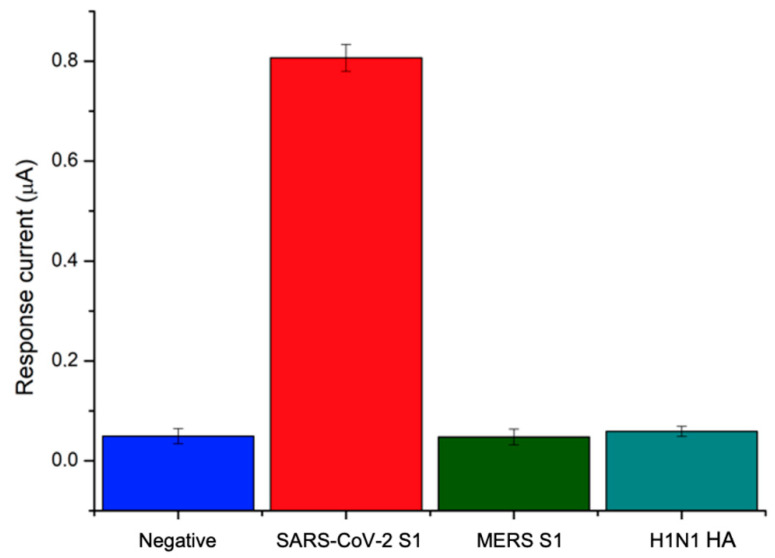
Specificity of the immunosensor’s response to MERS S1 and H1N1 HA antigens.

Furthermore, good precision was observed at the lower and upper level of the calibration curve, resulting in RSD values always lower than 10% (*n* = 3) over the explored concentration range. Since the nonspecific binding of an antibody with foreign molecules other than the target protein is a major issue in immunosensor development, the specificity of the response to the SARS-CoV-2 S1 protein was assessed by testing the immunosensor against different viral antigens, including Hemagglutinin from swine-origin influenza A (H1N1) virus and Spike Protein S1 from Middle East respiratory syndrome (MERS) Coronavirus. Indeed, these viruses give rise to similar symptoms with respect to Covid-19 disease and attack the respiratory system: H1N1 Hemmagglutinin is the virus surface protein involved in the binding of human receptor and recognized as a target by neutralizing antibodies, while MERS Spike protein S1 mediates viral binding to host cells and virus-cell membrane fusion, playing an essential role in MERS-CoV infection. The designed immunosensor was tested against both interfering proteins at a concentration of 40 ng/mL and the results were compared with negative and positive responses to SARS-CoV-2 S1. The results shown in Figure 6 demonstrate that neither H1N1 antigen nor MERS spike protein gave rise to a signal other than a negative response, thus demonstrating the remarkable specificity of the developed immunosensor.

### 3.3. Analysis of Whole Lentiviruses Expressing SARS-Cov-2 Spike Protein

To assess the ability of the immunosensor to detect and quantify whole SARS-CoV-2 virus, a third-generation replicating incompetent lentiviral vector pseudotyped with SARS-CoV-2 spike glycoprotein was used.

VTM was added to the lentivirus samples in order to simulate the collection of clinical specimens through nasopharyngeal swab. Using the immunosensing platform proposed, different lentivirus concentrations, ranging from 6.3∙10^7^ to 1.9∙10^6^ transducing units (TU)/mL, were tested. 

**Figure 7:**
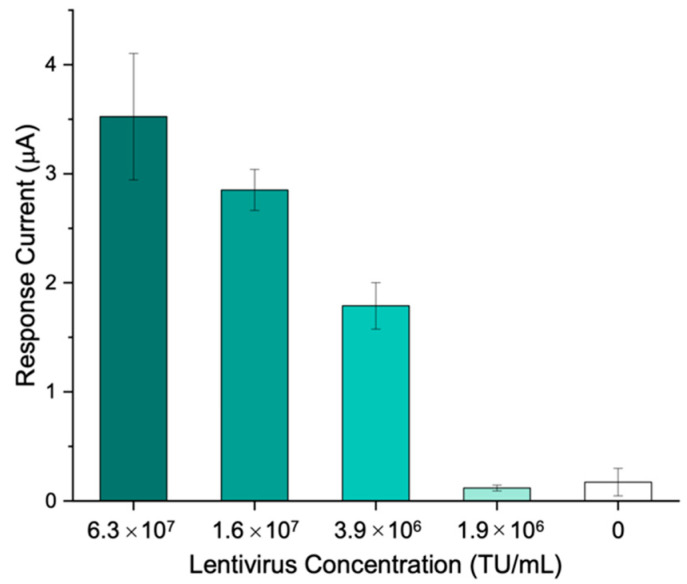
The electrochemical response of the designed immunosensor towards various whole SARS-CoV-2 replicating lentivirus concentrations.

The results show that a concentration of 3.9∙10^6^ TU/mL is sufficient to generate a signal which is significantly different (*p* < 0.001) from the blank obtained in the absence of the target, thus highlighting the applicability of the immunosensor to the whole virus detection (Figure 7).

A comparison between the developed immunosensor to determine SARS-CoV-2 Spike protein and other immunosensors is shown in Table 1.

The comparison highlights the advantages of our work over previous research, since it combines good sensitivity, specificity toward other viral antigens and applicability to the detection of whole virus with smart features that integrate ML-based data processing, as discussed below.

### 3.4. Use of Machine Learning for the Labelling of Positive/Negative SARS-CoV-2 S Antigen in VTM

The data generated by the developed immunosensor were investigated by applying ML techniques to quickly and automatically process the signal output of the immunosensor with the ultimate goal of classifying samples as positive or negative and improving the accuracy of the measurement. This feature is crucial for on-site detection of SARS-CoV-2 antigen and makes this solution a good candidate for PoCT contexts [4].

A set of 55 positive and 53 negative samples was acquired for a total of 108 data for training and validation purposes, reaching a dataset dimension comparable to previously reported studies dealing with SARS-CoV-2 pandemic [46,47]. A potential window ranging from −0.5 V to 0.1 V was set for the acquisition. Different SVM classifiers were evaluated with the main goal of selecting the best SVM kernel and optimal hyperparameters to achieve the highest classification accuracy computed as

(2) accuracy= (TP+TN)/(TP+TN+FP+FN)

where:

- *TP* are the True Positives (i.e., the samples that are classified in a class and actually belong to that class).
- *TN* are the True Negatives (i.e., the samples that are not classified in a class and actually do not belong to that class).
- *FP* are the False Positives (i.e., the samples that are classified in a class, but actually do not belong to that class).
- *FN* are the False Negatives (i.e., the samples that are not classified in a class, but actually belong to that class).

Data were processed using the MATLAB Classification Learner App and testing Linear, Quadratic, Cubic and Gaussian kernel functions, with and without optimization. Bayesian Optimization [48] was used and 100 iterations were considered. During optimization process, the MATLAB suite computes the best parameters to achieve maximum accuracy in the validation process. The parameters included in the optimization are the *Box Constraint Level* hyperparameter and the possible selection of the data standardization feature. 

The *Box Constraint* of an SVM classifier is a penalty factor that controls the maximum allowable misclassifications, which helps to prevent overfitting, i.e., the larger the *Box Constraint*, the fewer the support vectors in the trained model. In addition, selecting a higher value for this parameter results in a longer training time. To validate the trained model and ensure the generalizability of the results, a 10-fold cross-validation was performed.

Table 2 summarizes the results in terms of accuracy obtained in the validation phase.

It is noted that the best results were obtained using linear, quadratic and cubic kernels. In view of the future embedded implementation and C code generation with the MATLAB coder app, the linear solution was chosen, as the MCU firmware could benefit from its greater simplicity more than the polynomial version. In this case, the optimized model resulted in a value of 13.94 for the *Box Constraint Level* hyperparameter with data standardization. 

**Figure 8:**
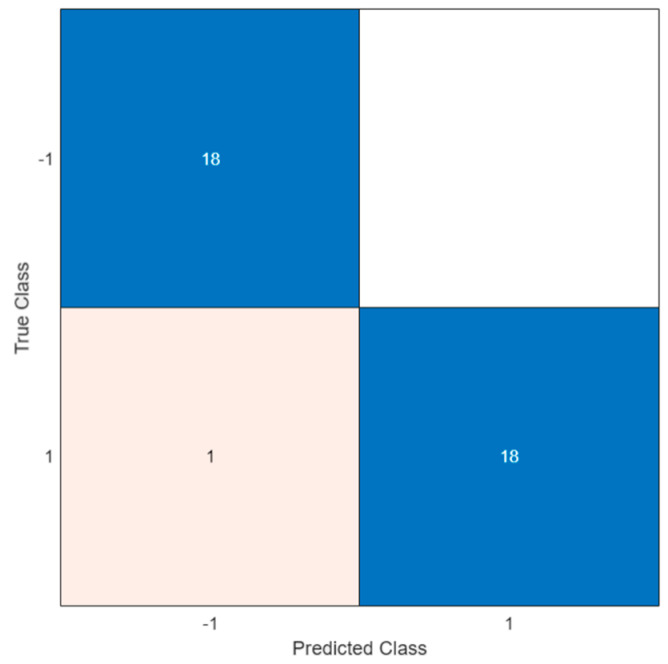
Confusion matrix obtained by sample classification according to ML model.

Therefore, this configuration was applied to verify the ability of the trained model to classify unknown data. A total of 19 positive and 18 negative solutions were analyzed, resulting in a test accuracy of 97.3%. Figure 8 illustrates the confusion matrix of the test.

In the confusion matrix, the positive and the negative classes are represented by the symbols ‘1′ and ‘−1′, respectively. As shown, a misclassification occurred in only one case; this led to a FN indicating the outcome where the model incorrectly predicted the negative class. However, all negative samples were correctly classified and no errors were observed (i.e., white square in the upper right of the matrix).

To evaluate the goodness of the results obtained, a comparison with a traditional semi-quantitative analysis was performed. To this end, data acquired with the potentiostat were automatically processed to measure the DPV peak current and compare it with the assessed LOD threshold to decide whether the sample was positive or negative [19]. To overcome the baseline drift [23] and correctly calculate the peak current, a baseline has to be estimated in advance. To this end, 30 relative minimum points were identified, and linear interpolation was applied to estimate the baseline. Based on a LOD signal of 0.3 µA, an accuracy of 83% was obtained. It is worth noting that this relatively low accuracy was due to the high output current from the potentiostat which occurred at the lower end of the potential range explored. Indeed, the accuracy of the threshold-based algorithm was severely affected by the DPV pattern. Better accuracy could be achieved with a narrower potential window. By contrast, the ML approach was not affected by this issue. Therefore, ML techniques show improved performance and allow baseline drift to be automatically overcome, thus simplifying the data preprocessing during the testing phase.

It is worth pointing out that the ML algorithm can be easily integrated into the cloud-based portable Wi-Fi device already described in [41]. The approach proposed in our previous work relies on custom hardware designed with performance comparable with commercial bench-top instruments, the ThingSpeak cloud service for data storage and analytics, and on a custom web application for remote control and data sharing with users, physician and other stakeholders. The smart potentiostat was designed to ensure maximum flexibility and reconfigurability and to support the implementation of various processing techniques. Therefore, the ML approach presented in this work can be easily integrated by performing the ML training phase on the cloud (e.g., Thingspeak), without relying on any additional external device. Moreover, if needed, the C code of the trained classifier can be generated and included in the device firmware to perform sample labelling directly on board. Compared to other solution presented in the literature [8,49,50], both the training and test phases can be processed autonomously (either on board or on the cloud platform), improving the ease of use and portability of the device, thus promoting its introduction in home and PoCT environments outside a clinical setting.

## 4. Conclusions

In the present work, ML algorithms were successfully applied to process data acquired with a smart and portable immunosensor aimed at antigenic tests for the detection and quantification of the SARS-CoV-2 spike protein. Optimization procedures were applied to the assay protocol by means of full factorial experimental design. As for ML algorithm, different SVM classifiers were evaluated, resulting in an excellent classification of positive and negative samples, i.e., a test accuracy of 97.3%.

The fitness for purpose of the sensing device developed for the antigen test was demonstrated by the validation in VTM matrix, conventionally used for the extraction of nasopharyngeal swabs, even with whole third-generation replicating incompetent lentiviral vector pseudotyped with SARS-CoV-2 spike glycoprotein. In addition, the use of monoclonal antibody receptor allowed us to achieve outstanding specificity with respect to other viral antigens associated with syndromes with overlapping symptoms, such as H1N1 and MERS S1, which may be present in real specimens.

The protocol for carrying out the electrochemical immunoassay is very fast and simple, requiring a single one-hour sample incubation on the SPE surface functionalized with monoclonal antibodies specific for SARS-CoV-2 S1 subunit. The sample must only be previously mixed with a solution containing all the reagents.

For these reasons, the developed smart immunosensor combines the ease of use of the LFIA strip test with the unique performance of electrochemical devices in terms of specificity, sensitivity and accuracy, with the added value of the quantitative response, not allowed by the LFIA strip tests.

Finally, the unique features of the portable and smart potentiostat enabled the implementation of all the acquisition and processing actions on a low-cost, low-power MCU platform with benefits in terms of flexibility, ease of use and long battery life. Nonetheless, future work is needed to develop and characterize a fully edge solution.

## Figures and Tables

**Table 1:** Comparison of the performance of the developed immunosensor with other electrochemical immunosensors for SARS-CoV-2 spike protein.

Sensing Approach	Lod	Loq	Whole Virus Detection	Specificity vs. Other Viral Antigens	Smart Features	References
ACE2 receptor covalently immobilized on gold nanoparticles modified laser-scribed graphene with label-free detection	5.14 ng/mL	N.D.	Yes	Moderate	Yes	[8]
Anti-spike IgG immobilized on graphene electrodes with label-free detection	20 µg/mL	N.D.	Yes	No	No	[34]
Anti-spike IgG immobilized on Cu_2_O nanocubes modified carbon electrodes with impedimetric detection	0.04 fg/mL	N.D.	Yes	Yes	No	[35]
Anti-spike IgG immobilized on graphene oxide modified carbon electrodes with label-free voltametric detection	1 ag/mL	N.D.	Yes	Yes	No	[36]
Anti-spike IgM immobilized on graphene oxide modified paper pads with label-free voltametric detection	0.11 ng/mL	N.D.	No	No	Yes	[37]
Receptor-free cobalt functionalized TiO_2_ nanotubes platform with amperometric detection	14 nM	N.D.	No	No	No	[38]
Sandwich based on monoclonal/polyclonal anti-spike IgGs immobilized on magnetic beads with enzyme-labelled voltametric detection	19 ng/mL	N.D.	Yes	Yes	No	[39]
Sandwich based on monoclonal/polyclonal anti-spike IgMs immobilized on gold nanoparticles modified carbon electrodes with enzyme-labelled voltametric detection	12 ng/mL	40 ng/mL	Yes	Yes	Yes	This study

N.D. = Not Declared.

**Table 2:** Accuracy values obtained with ML based on different SVM classifiers.

Kernel Function	Optimization	Accuracy
Linear	No optimization Bayesian Optimization	94.4% 99.1%
Quadratic	No optimization Bayesian Optimization	93.5% 99.1%
Cubic	No optimization Bayesian Optimization	94.4% 99.1%
Gaussian	No optimization Bayesian Optimization	86.1% 95.4%

## Data Availability

The data is available upon reasonable request from the corresponding author.

## Supplementary Materials

The following supporting information can be downloaded at: https://www.mdpi.com/article/10.3390/bios12060426/s1, Figure S1: Signals recorded in presence and absence of mA-S1.
