# Supplementary material for: Rapid Quantification of SARS-Cov-2 Spike Protein Enhanced with a Machine Learning Technique Integrated in a Smart and Portable Immunosensor
Source: Biosensors (Basel). 2022 Jun 17;12(6):426. doi: 10.3390/bios12060426 (PMC9220900; doi:10.3390/bios12060426)
Supplement: Supplementary file 1 [file biosensors-12-00426-s001.zip › biosensors-1748931-supplementary.pdf]

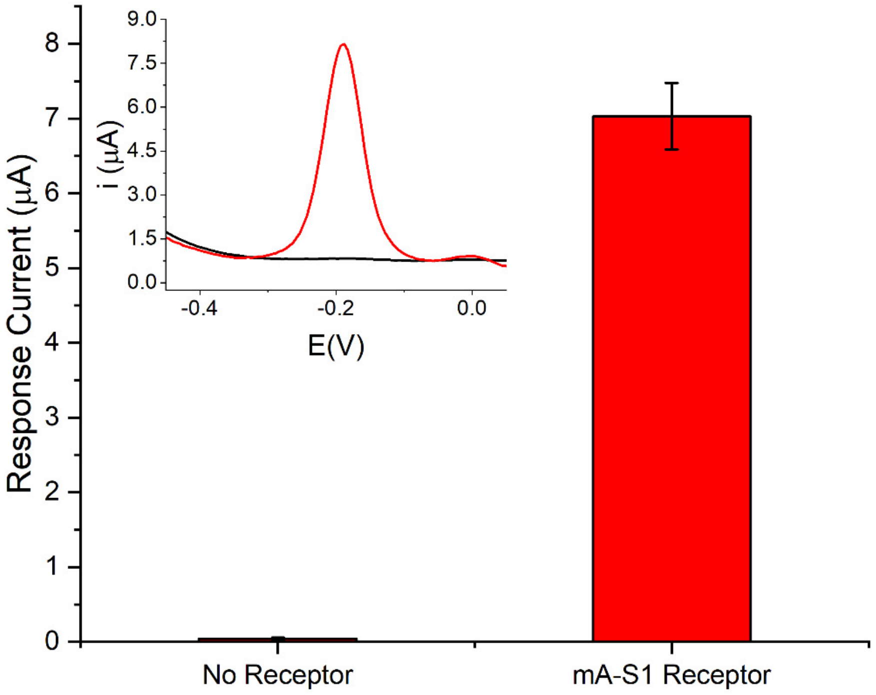

Figure S1. Signals recorded incubating incubating a 15 μg/mL solution of SARS-CoV-2 S1 containing also pA-S1 and GAR-AP at 10 and 2 μg/mL, respectively, on GNP-SPEs functionalized (right) and not functionalized (left) with mA-S1 receptor. Inset: DPV voltammograms recorded in the presence (red) and in the absence (black) of mA-S1 receptor on GNP-SPEs.
